# Genome-wide association study of germline variants and breast cancer-specific mortality

**DOI:** 10.1038/s41416-019-0393-x

**Published:** 2019-02-21

**Authors:** Maria Escala-Garcia, Qi Guo, Thilo Dörk, Sander Canisius, Renske Keeman, Joe Dennis, Jonathan Beesley, Julie Lecarpentier, Manjeet K. Bolla, Qin Wang, Jean Abraham, Irene L. Andrulis, Hoda Anton-Culver, Volker Arndt, Paul L. Auer, Matthias W. Beckmann, Sabine Behrens, Javier Benitez, Marina Bermisheva, Leslie Bernstein, Carl Blomqvist, Bram Boeckx, Stig E. Bojesen, Bernardo Bonanni, Anne-Lise Børresen-Dale, Hiltrud Brauch, Hermann Brenner, Adam Brentnall, Louise Brinton, Per Broberg, Ian W. Brock, Sara Y. Brucker, Barbara Burwinkel, Carlos Caldas, Trinidad Caldés, Daniele Campa, Federico Canzian, Angel Carracedo, Brian D. Carter, Jose E. Castelao, Jenny Chang-Claude, Stephen J. Chanock, Georgia Chenevix-Trench, Ting-Yuan David Cheng, Suet-Feung Chin, Christine L. Clarke, NBCS Collaborators, Emilie Cordina-Duverger, Fergus J. Couch, David G. Cox, Angela Cox, Simon S. Cross, Kamila Czene, Mary B. Daly, Peter Devilee, Janet A. Dunn, Alison M. Dunning, Lorraine Durcan, Miriam Dwek, Helena M. Earl, Arif B. Ekici, A. Heather Eliassen, Carolina Ellberg, Christoph Engel, Mikael Eriksson, D. Gareth Evans, Jonine Figueroa, Dieter Flesch-Janys, Henrik Flyger, Marike Gabrielson, Manuela Gago-Dominguez, Eva Galle, Susan M. Gapstur, Montserrat García-Closas, José A. García-Sáenz, Mia M. Gaudet, Angela George, Vassilios Georgoulias, Graham G. Giles, Gord Glendon, David E. Goldgar, Anna González-Neira, Grethe I. Grenaker Alnæs, Mervi Grip, Pascal Guénel, Lothar Haeberle, Eric Hahnen, Christopher A. Haiman, Niclas Håkansson, Per Hall, Ute Hamann, Susan Hankinson, Elaine F. Harkness, Patricia A. Harrington, Steven N. Hart, Jaana M. Hartikainen, Alexander Hein, Peter Hillemanns, Louise Hiller, Bernd Holleczek, Antoinette Hollestelle, Maartje J. Hooning, Robert N. Hoover, John L. Hopper, Anthony Howell, Guanmengqian Huang, Keith Humphreys, David J. Hunter, Wolfgang Janni, Esther M. John, Michael E. Jones, Arja Jukkola-Vuorinen, Audrey Jung, Rudolf Kaaks, Maria Kabisch, Katarzyna Kaczmarek, Michael J. Kerin, Sofia Khan, Elza Khusnutdinova, Johanna I. Kiiski, Cari M. Kitahara, Julia A. Knight, Yon-Dschun Ko, Linetta B. Koppert, Veli-Matti Kosma, Peter Kraft, Vessela N. Kristensen, Ute Krüger, Tabea Kühl, Diether Lambrechts, Loic Le Marchand, Eunjung Lee, Flavio Lejbkowicz, Lian Li, Annika Lindblom, Sara Lindström, Martha Linet, Jolanta Lissowska, Wing-Yee Lo, Sibylle Loibl, Jan Lubiński, Michael P. Lux, Robert J. MacInnis, Melanie Maierthaler, Tom Maishman, Enes Makalic, Arto Mannermaa, Mehdi Manoochehri, Siranoush Manoukian, Sara Margolin, Maria Elena Martinez, Dimitrios Mavroudis, Catriona McLean, Alfons Meindl, Pooja Middha, Nicola Miller, Roger L. Milne, Fernando Moreno, Anna Marie Mulligan, Claire Mulot, Rami Nassir, Susan L. Neuhausen, William T. Newman, Sune F. Nielsen, Børge G. Nordestgaard, Aaron Norman, Håkan Olsson, Nick Orr, V. Shane Pankratz, Tjoung-Won Park-Simon, Jose I. A. Perez, Clara Pérez-Barrios, Paolo Peterlongo, Christos Petridis, Mila Pinchev, Karoliona Prajzendanc, Ross Prentice, Nadege Presneau, Darya Prokofieva, Katri Pylkäs, Brigitte Rack, Paolo Radice, Dhanya Ramachandran, Gadi Rennert, Hedy S. Rennert, Valerie Rhenius, Atocha Romero, Rebecca Roylance, Emmanouil Saloustros, Elinor J. Sawyer, Daniel F. Schmidt, Rita K. Schmutzler, Andreas Schneeweiss, Minouk J. Schoemaker, Fredrick Schumacher, Lukas Schwentner, Rodney J. Scott, Christopher Scott, Caroline Seynaeve, Mitul Shah, Jacques Simard, Ann Smeets, Christof Sohn, Melissa C. Southey, Anthony J. Swerdlow, Aline Talhouk, Rulla M. Tamimi, William J. Tapper, Manuel R. Teixeira, Maria Tengström, Mary Beth Terry, Kathrin Thöne, Rob A. E. M. Tollenaar, Ian Tomlinson, Diana Torres, Thérèse Truong, Constance Turman, Clare Turnbull, Hans-Ulrich Ulmer, Michael Untch, Celine Vachon, Christi J. van Asperen, Ans M. W. van den Ouweland, Elke M. van Veen, Camilla Wendt, Alice S. Whittemore, Walter Willett, Robert Winqvist, Alicja Wolk, Xiaohong R. Yang, Yan Zhang, Douglas F. Easton, Peter A. Fasching, Heli Nevanlinna, Diana M. Eccles, Paul D. P. Pharoah, Marjanka K. Schmidt

**Affiliations:** 1 The Netherlands Cancer Institute, Antoni van Leeuwenhoek Hospital, Division of Molecular Pathology, Amsterdam, The Netherlands; 2 University of Cambridge, Cardiovascular Epidemiology Unit, Department of Public Health and Primary Care, Cambridge, UK; 3 Hannover Medical School, Gynaecology Research Unit, Hannover, Germany; 4 The Netherlands Cancer Institute, Antoni van Leeuwenhoek Hospital, Division of Molecular Carcinogenesis, Amsterdam, The Netherlands; 5 University of Cambridge, Centre for Cancer Genetic Epidemiology, Department of Public Health and Primary Care, Cambridge, UK; 6 QIMR Berghofer Medical Research Institute, Department of Genetics and Computational Biology, Brisbane, Queensland Australia; 7 University of Cambridge, Centre for Cancer Genetic Epidemiology, Department of Oncology, Cambridge, UK; 8 Cambridge Experimental Cancer Medicine Centre, Cambridge, UK; 9 University of Cambridge NHS Foundation Hospitals, Cambridge Breast Unit and NIHR Cambridge Biomedical Research Centre, Cambridge, UK; 10 Lunenfeld-Tanenbaum Research Institute of Mount Sinai Hospital, Fred A. Litwin Center for Cancer Genetics, Toronto, ON Canada; 11 University of Toronto, Department of Molecular Genetics, Toronto, ON Canada; 12 University of California Irvine, Department of Epidemiology, Genetic Epidemiology Research Institute, Irvine, CA USA; 13 German Cancer Research Center (DKFZ), Division of Clinical Epidemiology and Aging Research, Heidelberg, Germany; 14 Fred Hutchinson Cancer Research Center, Cancer Prevention Program, Seattle, WA USA; 15 University of Wisconsin-Milwaukee, Zilber School of Public Health, Milwaukee, WI USA; 16 University Hospital Erlangen, Friedrich-Alexander-University Erlangen-Nuremberg, Department of Gynecology and Obstetrics, Comprehensive Cancer Center ER-EMN, Erlangen, Germany; 17 German Cancer Research Center (DKFZ), Division of Cancer Epidemiology, Heidelberg, Germany; 18 Spanish National Cancer Research Centre (CNIO), Human Cancer Genetics Programme, Madrid, Spain; 19 Biomedical Network on Rare Diseases (CIBERER), Madrid, Spain; 20 Ufa Scientific Center of Russian Academy of Sciences, Institute of Biochemistry and Genetics, Ufa, Russia; 21 Beckman Research Institute of City of Hope, Department of Population Sciences, Duarte, CA USA; 22 University of Helsinki, Department of Oncology, Helsinki University Hospital, Helsinki, Finland; 23 Örebro University Hospital, Department of Oncology, Örebro, Sweden; 24 VIB, VIB Center for Cancer Biology, Leuven, Belgium; 25 University of Leuven, Laboratory for Translational Genetics, Department of Human Genetics, Leuven, Belgium; 26 Copenhagen University Hospital, Copenhagen General Population Study, Herlevand Gentofte Hospital, Herlev, Denmark; 27 Copenhagen University Hospital, Department of Clinical Biochemistry, Herlev and Gentofte Hospital, Herlev, Denmark; 28 University of Copenhagen, Faculty of Health and Medical Sciences, Copenhagen, Denmark; 29 Division of Cancer Prevention and Genetics, IEO, European Institute of Oncology IRCCS Milan, Milan, 20141 Italy; 30 Oslo University Hospital-Radiumhospitalet, Department of Cancer Genetics, Institute for Cancer Research, Oslo, Norway; 31 University of Oslo, Institute of Clinical Medicine, Faculty of Medicine, Oslo, Norway; 32 Department of Research, Vestre Viken Hospital, Drammen, Norway; Section for Breast- and Endocrine Surgery, Department of Cancer, Division of Surgery, Cancer and Transplantation Medicine, Oslo University Hospital-Ullevål, Oslo, Norway; 33 Department of Radiology and Nuclear Medicine, Oslo University Hospital, Oslo, Norway; 34 Department of Pathology at Akershus University hospital, Lørenskog, Norway; 35 Department of Tumor Biology, Institute for Cancer Research, Oslo University Hospital, Oslo, Norway; 36 Department of Oncology, Division of Surgery and Cancer and Transplantation Medicine, Oslo University Hospital-Radiumhospitalet, Oslo, Norway; 37 National Advisory Unit on Late Effects after Cancer Treatment, Department of Oncology, Oslo University Hospital, Oslo, Norway; 38 Department of Oncology, Akershus University Hospital, Lørenskog, Norway; 39 Breast Cancer Research Consortium, Oslo University Hospital, Oslo, Norway; 40 Dr. Margarete Fischer-Bosch-Institute of Clinical Pharmacology, Stuttgart, Germany; 41 University of Tübingen, Tübingen, Germany; 42 German Cancer Research Center (DKFZ), German Cancer Consortium (DKTK), Heidelberg, Germany; 43 German Cancer Research Center (DKFZ) and National Center for Tumor Diseases (NCT), Division of Preventive Oncology, Heidelberg, Germany; 44 Queen Mary University of London, Centre for Cancer Prevention, Wolfson Institute of Preventive Medicine, London, UK; 45 National Cancer Institute, Division of Cancer Epidemiology and Genetics, Bethesda, MD USA; 46 Lund University, Department of Cancer Epidemiology, Clinical Sciences, Lund, Sweden; 47 University of Sheffield, Sheffield Institute for Nucleic Acids (SInFoNiA), Department of Oncology and Metabolism, Sheffield, UK; 48 University of Tübingen, Department of Gynecology and Obstetrics, Tübingen, Germany; 49 University of Heidelberg, Department of Obstetrics and Gynecology, Heidelberg, Germany; 50 German Cancer Research Center (DKFZ), Molecular Epidemiology Group, C080 Heidelberg, Germany; 51 The Institute of Cancer Research, Section of Cancer Genetics, London, UK; 52 Instituto de Investigación Sanitaria San Carlos (IdISSC), Centro Investigación Biomédica en Red de Cáncer (CIBERONC), Medical Oncology Department, Hospital Cl’nico San Carlos, Madrid, Spain; 53 University of Pisa, Department of Biology, Pisa, Italy; 54 Instituto de Investigación Sanitaria de Santiago de Compostela (IDIS), Genomic Medicine Group, Galician Foundation of Genomic Medicine, SERGAS, Santiago de Compostela, Spain; 55 Universidad de Santiago de Compostela, Centro de Investigación en Red de Enfermedades Raras (CIBERER), Santiago De Compostela, Spain; 56 King Abdulaziz University, Center of Excellence in Genomic Medicine, Jeddah, Kingdom of Saudi Arabia; 57 American Cancer Society, Epidemiology Research Program, Atlanta, GA USA; 58 Instituto de Investigación Sanitaria Galicia Sur (IISGS), Xerencia de Xestion Integrada de Vigo-SERGAS, Oncology and Genetics Unit, Vigo, Spain; 59 University Medical Center Hamburg-Eppendorf, Cancer Epidemiology Group, University Cancer Center Hamburg (UCCH), Hamburg, Germany; 60 Roswell Park Cancer Institute, Division of Cancer Prevention and Control, Buffalo, NY USA; 61 University of Cambridge, Cancer Research UK Cambridge Institute, Cambridge, UK; 62 University of Sydney, Westmead Institute for Medical Research, Sydney, NSW Australia; 63 INSERM, University Paris-Sud, University Paris-Saclay, Cancer & Environment Group, Center for Research in Epidemiology and Population Health (CESP), Villejuif, France; 64 Mayo Clinic, Department of Laboratory Medicine and Pathology, Rochester, MN USA; 65 Imperial College London, Department of Epidemiology and Biostatistics, School of Public Health, London, UK; 66 Cancer Research Center of Lyon, INSERM U1052 Lyon, France; 67 University of Sheffield, Academic Unit of Pathology, Department of Neuroscience, Sheffield, UK; 68 Karolinska Institutet, Department of Medical Epidemiology and Biostatistics, Stockholm, Sweden; 69 Fox Chase Cancer Center, Department of Clinical Genetics, Philadelphia, PA USA; 70 Leiden University Medical Center, Department of Pathology, Leiden, The Netherlands; 71 Leiden University Medical Center, Department of Human Genetics, Leiden, The Netherlands; 72 University of Warwick, Warwick Clinical Trials Unit, Coventry, UK; 73 University of Southampton, Southampton Clinical Trials Unit, Faculty of Medicine, Southampton, UK; 74 University of Southampton, Cancer Sciences Academic Unit, Faculty of Medicine, Southampton, UK; 75 University of Westminster, Department of Biomedical Sciences, Faculty of Science and Technology, London, UK; 76 University of Cambridge, Department of Oncology, Cambridge, UK; 77 Friedrich-Alexander University Erlangen-Nuremberg, Comprehensive Cancer Center Erlangen-EMN, Institute of Human Genetics, University Hospital Erlangen, Erlangen, Germany; 78 Harvard Medical School, Channing Division of Network Medicine, Department of Medicine, Brigham and Women’s Hospital, Boston, MA USA; 79 Harvard T.H. Chan School of Public Health, Department of Epidemiology, Boston, MA USA; 80 University of Leipzig, Institute for Medical Informatics, Statistics and Epidemiology, Leipzig, Germany; 81 University of Leipzig, LIFE - Leipzig Research Centre for Civilization Diseases, Leipzig, Germany; 82 University of Manchester, Manchester Academic Health Science Centre, Division of Evolution and Genomic Medicine, School of Biological Sciences, Faculty of Biology, Medicine and Health, Manchester, UK; 83 St Marys Hospital, Central Manchester University Hospitals NHS Foundation Trust, Manchester Academic Health Science Centre, Manchester Centre for Genomic Medicine, Manchester, UK; 84 The University of Edinburgh Medical School, Usher Institute of Population Health Sciences and Informatics, Edinburgh, UK; 85 Cancer Research UK Edinburgh Centre, Edinburgh, UK; 86 University Medical Centre Hamburg-Eppendorf, Institute for Medical Biometrics and Epidemiology, Hamburg, Germany; 87 University Medical Centre Hamburg-Eppendorf, Department of Cancer Epidemiology, Clinical Cancer Registry, Hamburg, Germany; 88 Copenhagen University Hospital, Department of Breast Surgery, Herlev and Gentofte Hospital, Herlev, Denmark; 89 University of California San Diego, Moores Cancer Center, La Jolla, CA USA; 90 Institute of Cancer Research, Division of Genetics and Epidemiology, London, UK; 91 The Institute of Cancer Research, Division of Genetics and Epidemiology, London, UK; 92 The Royal Marsden NHS Foundation Trust, Cancer Genetics Unit, London, UK; 93 University Hospital of Heraklion, Department of Medical Oncology, Heraklion, Greece; 94 Cancer Council Victoria, Cancer Epidemiology & Intelligence Division, Melbourne, VIC Australia; 95 The University of Melbourne, Melbourne School of Population and Global Health, Centre for Epidemiology and Biostatistics, Melbourne, VIC Australia; 96 Monash University, Department of Epidemiology and Preventive Medicine, Melbourne, VIC Australia; 97 Huntsman Cancer Institute, University of Utah School of Medicine, Department of Dermatology, Salt Lake City, UT USA; 98 University of Oulu, Department of Surgery, Oulu University Hospital, Oulu, Finland; 99 Friedrich-Alexander University Erlangen-Nuremberg, Comprehensive Cancer Centre Erlangen-EMN, Department of Gynaecology and Obstetrics, University Hospital Erlangen, Erlangen, Germany; 100 University Hospital of Cologne, Centre for Hereditary Breast and Ovarian Cancer, Cologne, Germany; 101 University of Cologne, Centre for Molecular Medicine Cologne (CMMC), Cologne, Germany; 102 University of Southern California, Department of Preventive Medicine, Keck School of Medicine, Los Angeles, CA USA; 103 Karolinska Institutet, Institute of Environmental Medicine, Stockholm, Sweden; 104 South General Hospital, Department of Oncology, Stockholm, Sweden; 105 German Cancer Research Centre (DKFZ), Molecular Genetics of Breast Cancer, Heidelberg, Germany; 106 University of Massachusetts, Amherst, Department of Biostatistics & Epidemiology, Amherst, MA USA; 107 University of Manchester, Manchester Academic Health Science Centre, Division of Informatics, Imaging and Data Sciences, Faculty of Biology, Medicine and Health, Manchester, UK; 108 Wythenshawe Hospital, Manchester University NHS Foundation Trust, Nightingale Breast Screening Centre, Manchester, UK; 109 Manchester University NHS Foundation Trust, Manchester Academic Health Science Centre, NIHR Manchester Biomedical Research Unit, Manchester, UK; 110 Mayo Clinic, Department of Health Sciences Research, Rochester, MN USA; 111 University of Eastern Finland, Translational Cancer Research Area, Kuopio, Finland; 112 University of Eastern Finland, Institute of Clinical Medicine, Pathology and Forensic Medicine, Kuopio, Finland; 113 Kuopio University Hospital, Imaging Centre, Department of Clinical Pathology, Kuopio, Finland; 114 Saarland Cancer Registry, Saarbruecken, Germany; 115 Erasmus MC Cancer Institute, Department of Medical Oncology, Family Cancer Clinic, Rotterdam, The Netherlands; 116 University of Manchester, Institute of Cancer studies, Manchester, UK; 117 Harvard T.H. Chan School of Public Health, Program in Genetic Epidemiology and Statistical Genetics, Boston, MA USA; 118 University of Oxford, Nuffield Department of Population Health, Oxford, UK; 119 Cancer Research UK Edinburgh Centre, Edinburgh, UK; 120 Cancer Prevention Institute of California, Department of Epidemiology, Fremont, CA USA; 121 Stanford University School of Medicine, Department of Health Research and Policy - Epidemiology, Stanford, CA USA; 122 Stanford University School of Medicine, Department of Biomedical Data Science, Stanford, CA USA; 123 Tampere University Hospital, Department of Oncology, Tampere, Finland; 124 Pomeranian Medical University, Department of Genetics and Pathology, Szczecin, Poland; 125 National University of Ireland, Surgery, School of Medicine, Galway, Ireland; 126 University of Helsinki, Department of Obstetrics and Gynaecology, Helsinki University Hospital, Helsinki, Finland; 127 Bashkir State University, Department of Genetics and Fundamental Medicine, Ufa, Russia; 128 National Cancer Institute, Radiation Epidemiology Branch, Division of Cancer Epidemiology and Genetics, Bethesda, MD USA; 129 Lunenfeld-Tanenbaum Research Institute, Sinai Health System, Prosserman Centre for Population Health Research, Toronto, ON Canada; 130 University of Toronto, Division of Epidemiology, Dalla Lana School of Public Health, Toronto, ON Canada; 131 Johanniter Krankenhaus, Department of Internal Medicine, Evangelische Kliniken Bonn gGmbH, Bonn, Germany; 132 Erasmus MC Cancer Institute, Department of Surgical Oncology, Family Cancer Clinic, Rotterdam, The Netherlands; 133 University of Hawaii Cancer Center, Epidemiology Program, Honolulu, HI USA; 134 Carmel Medical Center and Technion Faculty of Medicine, Clalit National Cancer Control Center, Haifa, Israel; 135 Tianjin Medical University Cancer Institute and Hospital, Department of Epidemiology, Tianjin, China; 136 Karolinska Institutet, Department of Molecular Medicine and Surgery, Stockholm, Sweden; 137 University of Washington School of Public Health, Department of Epidemiology, Seattle, WA USA; 138 Fred Hutchinson Cancer Research Center, Public Health Sciences Division, Seattle, WA USA; 139 M. Sklodowska-Curie Cancer Centre, Oncology Institute, Department of Cancer Epidemiology and Prevention, Warsaw, Poland; 140 GmbH, German Breast Group, Neu Isenburg, Germany; 141 Fondazione IRCCS (Istituto Di Ricovero e Cura a Carattere Scientifico) Istituto Nazionale dei Tumori (INT), Unit of Medical Genetics, Department of Medical Oncology and Haematology, Milan, Italy; 142 Karolinska Institutet, Department of Clinical Science and Education, Sšdersjukhuset, Stockholm, Sweden; 143 University of California San Diego, Department of Family Medicine and Public Health, La Jolla, CA USA; 144 The Alfred Hospital, Anatomical Pathology, Melbourne, VIC Australia; 145 Ludwig Maximilian University of Munich, Department of Gynaecology and Obstetrics, Munich, Germany; 146 University of Heidelberg, Faculty of Medicine, Heidelberg, Germany; 147 University of Toronto, Department of Laboratory Medicine and Pathobiology, Toronto, ON Canada; 148 University Health Network, Laboratory Medicine Program, Toronto, ON Canada; 149 INSERM UMR-S1147, Université Paris Sorbonne Cité, Paris, France; 150 University of California Davis, Department of Biochemistry and Molecular Medicine, Davis, CA USA; 151 Queen’s University Belfast, Centre for Cancer Research and Cell Biology, Belfast, Ireland UK; 152 University of New Mexico, University of New Mexico Health Sciences Center, Albuquerque, NM USA; 153 Hospital Monte Naranco, Servicio de Cirug’a General y Especialidades, Oviedo, Spain; 154 Hospital Universitario Puerta de Hierro, Medical Oncology Department, Madrid, Spain; 155 The FIRC (Italian Foundation for Cancer Research) Institute of Molecular Oncology, IFOM, Milan, Italy; 156 King’s College London, Research Oncology, Guy’s Hospital, London, UK; 157 University of Oulu, Laboratory of Cancer Genetics and Tumour Biology, Cancer and Translational Medicine Research Unit, Biocentre Oulu, Oulu, Finland; 158 Northern Finland Laboratory Centre Oulu, Laboratory of Cancer Genetics and Tumour Biology, Oulu, Finland; 159 Fondazione IRCCS (Istituto Di Ricovero e Cura a Carattere Scientifico) Istituto Nazionale dei Tumori (INT), Unit of Molecular Bases of Genetic Risk and Genetic Testing, Department of Research, Milan, Italy; 160 UCLH Foundation Trust, Department of Oncology, London, UK; 161 University Hospital of Larissa, Department of Oncology, Larissa, Greece; 162 University of Heidelberg, National Centre for Tumour Diseases, Heidelberg, Germany; 163 Case Western Reserve University, Department of Population and Quantitative Health Sciences, Cleveland, OH USA; 164 John Hunter Hospital, Division of Molecular Medicine, Pathology North, Newcastle, NSW Australia; 165 University of Newcastle, Discipline of Medical Genetics, School of Biomedical Sciences and Pharmacy, Faculty of Health, Callaghan, NSW Australia; 166 John Hunter Hospital, Hunter Medical Research Institute, Newcastle, NSW Australia; 167 University of Newcastle, Centre for Information Based Medicine, Callaghan, Newcastle, NSW Australia; 168 Centre Hospitalier Universitaire de Québec - Université Laval Research Centre, Genomics Centre, Québec City, QC Canada; 169 University Hospitals Leuven, Department of Surgical Oncology, Leuven, Belgium; 170 Monash University, Precision Medicine, School of Clinical Sciences at Monash Health, Clayton, Victoria, Australia; 171 The University of Melbourne, Department of Clinical Pathology, Melbourne, VIC Australia; 172 The Institute of Cancer Research, Division of Breast Cancer Research, London, UK; 173 BC Cancer Agency and University of British Columbia, British Columbia’s Ovarian Cancer Research (OVCARE) Program, Vancouver General Hospital, Vancouver, BC Canada; 174 University of British Columbia, Department of Pathology and Laboratory Medicine, Vancouver, BC Canada; 175 University of British Columbia, Department of Obstetrics and Gynaecology, Vancouver, BC Canada; 176 University of Southampton, Faculty of Medicine, Southampton, UK; 177 Portuguese Oncology Institute, Department of Genetics, Porto, Portugal; 178 University of Porto, Biomedical Sciences Institute (ICBAS), Porto, Portugal; 179 Kuopio University Hospital, Cancer Centre, Kuopio, Finland; 180 University of Eastern Finland, Institute of Clinical Medicine, Oncology, Kuopio, Finland; 181 Columbia University, Department of Epidemiology, Mailman School of Public Health, New York, NY USA; 182 Leiden University Medical Centre, Department of Surgery, Leiden, The Netherlands; 183 University of Birmingham, Institute of Cancer and Genomic Sciences, Birmingham, UK; 184 University of Oxford, Wellcome Trust Centre for Human Genetics and Oxford NIHR Biomedical Research Centre, Oxford, UK; 185 Pontificia Universidad Javeriana, Institute of Human Genetics, Bogota, Colombia; 186 Frauenklinik der Stadtklinik Baden-Baden, Baden-Baden, Germany; 187 Helios Clinics Berlin-Buch, Department of Gynaecology and Obstetrics, Berlin, Germany; 188 Leiden University Medical Centre, Department of Clinical Genetics, Leiden, The Netherlands; 189 Erasmus University Medical Centre, Department of Clinical Genetics, Rotterdam, The Netherlands; 190 Karolinska Institutet, Department of Clinical Science and Education, Södersjukhuset, Stockholm, Sweden; 191 Harvard T.H. Chan School of Public Health, Department of Nutrition, Boston, MA USA; 192 Brigham and Women’s Hospital and Harvard Medical School, Channing Division of Network Medicine, Boston, MA USA; 193 Karolinska Institutet, Department of Environmental Medicine, Division of Nutritional Epidemiology, Stockholm, Sweden; 194 University of California at Los Angeles, David Geffen School of Medicine, Department of Medicine Division of Hematology and Oncology, Los Angeles, CA USA; 195 The Netherlands Cancer Institute - Antoni van Leeuwenhoek hospital, Division of Psychosocial Research and Epidemiology, Amsterdam, The Netherlands

**Keywords:** Breast cancer, Prognosis, Prognostic markers, Cancer genetics

## Abstract

**Background:**

We examined the associations between germline variants and breast cancer mortality using a large meta-analysis of women of European ancestry.

**Methods:**

Meta-analyses included summary estimates based on Cox models of twelve datasets using ~10.4 million variants for 96,661 women with breast cancer and 7697 events (breast cancer-specific deaths). Oestrogen receptor (ER)-specific analyses were based on 64,171 ER-positive (4116) and 16,172 ER-negative (2125) patients. We evaluated the probability of a signal to be a true positive using the Bayesian false discovery probability (BFDP).

**Results:**

We did not find any variant associated with breast cancer-specific mortality at *P* < 5 × 10^−8^. For ER-positive disease, the most significantly associated variant was chr7:rs4717568 (BFDP = 7%, *P* = 1.28 × 10^−7^, hazard ratio [HR] = 0.88, 95% confidence interval [CI] = 0.84–0.92); the closest gene is *AUTS2*. For ER-negative disease, the most significant variant was chr7:rs67918676 (BFDP = 11%, *P* = 1.38 × 10^−7^, HR = 1.27, 95% CI = 1.16–1.39); located within a long intergenic non-coding RNA gene (AC004009.3), close to the *HOXA* gene cluster.

**Conclusions:**

We uncovered germline variants on chromosome 7 at BFDP < 15% close to genes for which there is biological evidence related to breast cancer outcome. However, the paucity of variants associated with mortality at genome-wide significance underpins the challenge in providing genetic-based individualised prognostic information for breast cancer patients.

## BACKGROUND

Breast cancer is the most common cancer in the Western world and accounts for 15% of cancer-related deaths in women, with about 522,000 deaths worldwide in 2012.^1^ Survival after a diagnosis of breast cancer varies considerably between patients even with closely matching tumour characteristics. Models that predict the likelihood of survival after breast cancer treatment use tumour and treatment data, but currently do not take host factors into account. The identification of prognostic and predictive biomarkers inherent in the germline of the patients rather than the tumour could pinpoint mechanisms of tumour progression and help with treatment stratification to increase therapeutic benefit. Such markers include inherited genetic variation, as there is evidence for heritability of breast cancer-specific mortality in affected first-degree relatives.^2–5^ Germline variation may affect prognosis by affecting tumour biology, since such variants are known to be associated with risk of specific breast tumour subtypes, particularly those defined by hormone receptor status, and have different outcomes.^6–8^ Germline genotype could also affect the efficacy of adjuvant drug therapies^9,10^ or might condition the host tumour environment via vascularisation,^11,12^ metastatic pattern,^13,14^ stroma–tumour interaction^15,16^ and immune surveillance.^17,18^

The association between common germline genetic variation and breast cancer-specific mortality has been examined in many candidate gene studies,^5,9,14,19–36^ as well as in moderate-sized genome-wide association studies (GWAS).^37–41^ However, it has been difficult link GWAS results to plausible candidate genes and few have been convincingly replicated.^29,42^ Large studies with long follow-up and reliable data on known prognostic factors are required if novel alleles associated with prognosis in breast cancer are to be identified at a level of genome-wide significance. In the present work, we pooled genotype data from multiple breast cancer GWAS discovery and replication efforts^43,44^ with new genotype data obtained from a large breast cancer series genotyped using the OncoArray chip.^45,46^ We examined associations with risk of breast cancer-specific mortality in a total of 96,661 breast cancer patients with survival time data. We then investigated the potential functional role of the selected variants by predicting possible target genes.

## Materials and methods

### Breast cancer patient samples

We included data from twelve datasets (*n* = 96,661) in which multiple breast cancer patient cohorts were genotyped by a variety of arrays providing genome-wide coverage of common variants. An overview of the datasets with specification of the arrays used is given in Supplementary Table 1. Data from eight of these datasets have been used in previous analyses (*n* = 37,954).^44^ However, the Collaborative Oncological Gene-Environment Study (COGS) dataset from the Breast Cancer Association Consortium (BCAC) was updated to include additional follow-up and death events and additional genotype data, increasing the number of events and samples to a total of *n* = 29,959 patients. Two new datasets, the BCAC OncoArray and the SUCCESS A trial, comprising 58,027 samples, were added for the current analyses.

The OncoArray is a custom Illumina genotyping array designed by the Genetic Associations and Mechanisms in Oncology (GAME-ON) consortium. It includes 533,000 variants of which 260,660 form a GWAS backbone, with the remainder being custom content, details of which have been described previously.^45^ The SUCCESS-A Study^47^ is a randomised phase III study of *n* = 3,299 breast cancer cases. Cases from the trial were genotyped using the Illumina Human OmniExpress array. We downloaded imputed genotypes from dbGaP (data reference 6266).

COGS samples that were also genotyped on the OncoArray were removed from the COGS dataset (*n* = 14,426). Female patients with invasive breast cancer diagnosed at age > 18 years, and with follow-up data available were included in the analyses. BCAC data from freeze 8 was used, in which 873 COGS samples with unknown breast cancer-specific mortality status were excluded from the analyses. All stages of cancer, including metastatic, were used in the analysis. Some individual studies applied additional selection criteria such as young age or early breast cancer stage (Supplementary Table 2).

### Genotype and sample quality control, ancestry analysis and imputation

The genotype and sample quality control for the datasets have been described previously.^44,45,47,48^ Ancestry outliers for each dataset were identified by multidimensional scaling or LAMP^49^ on the basis of a set of unlinked variants and HapMap2 populations. Samples of European ancestry were retained for analyses.

Ten of the datasets were imputed using the reference panel from the 1000 Genomes Project in a two-stage procedure. The 1000 Genomes project Phase 3 (October 2014) release was used as the reference panel for all the datasets apart from SUCCESS-A, which used the Phase 1 release (March 2012). Imputation for CGEMS and BPC3 was performed using the programme MACH.^50^ Phased genotypes were first derived using SHAPEIT^51^ and IMPUTE2^52^ and then used to perform imputation on the phased data. The main analyses were based on variants that were imputed with imputation *r*^2^ > 0.3 and had minor allele frequency (MAF) > 0.01 in at least one of the datasets leading to ~10.4 million variants. To match the individual datasets in the meta-analysis we used the chromosome position. Variants were kept in the analysis as long as they were present in one of the studies. In those cases where there was ambiguity over the naming of the insertions and deletions, the MAF was used for further matching.

### Statistical and bioinformatic methods

Time-to-event was calculated from the date of diagnosis. For prevalent cases with study entry after diagnosis left truncation was applied, i.e., follow-up started at the date of study entry.^53^ Follow-up was right censored on the date of death, on the date last known alive if death did not occur, or at 15 years after diagnosis, whichever came first. We chose the 15 years cut-off because follow-up varied between studies and after that period follow-up data became scarce. Follow-up of the cohorts is illustrated in Kaplan Meier curves (Supplementary Figure 1).

The hazard ratios (HR) for the association of genotypes with breast cancer-specific mortality were estimated using Cox proportional hazards regression^54^ implemented in an in-house programme written in C++. Analysis of the CGEMS and BPC3 data was conducted using ProbABEL.^55^ The estimates of the individual studies were combined using an inverse-variance weighted meta-analysis. Since meta-analysis results based on the Wald test have been shown to be inflated for rare variants^56^ we recomputed the standard errors based on the likelihood ratio test statistic (see details in Supplementary methods), using the formula:

$${\mathrm{SE}} = {\mathrm{log}}\left( {{\mathrm{HR}}} \right){\mathrm{/sqrt}}\left( {{\mathrm{LRT}}} \right)$$

For each dataset we included as covariates a variable number of principal components (Supplementary Table 1) from the ancestry analysis as covariates in order to control for cryptic population substructure. The Cox models were stratified by country for the OncoArray dataset and by study for the COGS dataset. Statistical tests were performed for each variant by combining the results for all the datasets using a fixed-effects meta-analysis. Inflation of the test statistics (*λ*) was estimated by dividing the 45th percentile of the test statistic by 0.357 (the 45th percentile for a *χ*^2^ distribution on 1 degree of freedom). Analyses were carried out for all invasive breast cancer and for oestrogen receptor (ER)-positive and ER-negative disease separately.

To assess the probability of a variant being a false positive we used a Bayesian false discovery probability (BFDP)^57^ test based on the *P* value, a prior set to 0.0001 and an upper likely HR of 1.3.

To predict potential target genes, we used Bedtools v2.26 to intersect notable variants with genomic annotation data relevant to gene regulation activity in samples derived from breast tissue. We examined features including enhancers, promoters and transcription factor binding sites identified by the Roadmap^58^ and ENCODE^59^ Projects. Expression quantitative loci (eQTL) data from GTEx^60^ were queried for evidence of potential *cis*-regulatory activity.

## Results

**Fig. 1:**
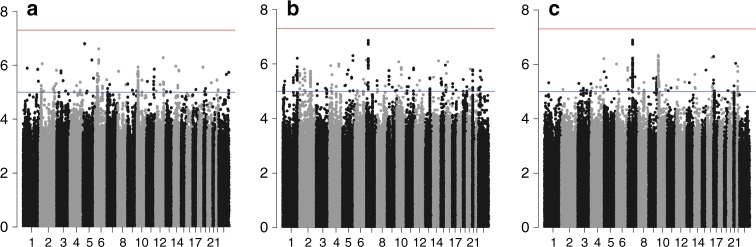
Association plot for the meta-analysis of the twelve datasets for breast cancer-specific mortality analyses (censored at 15 years) for **a** all breast tumours (censored at 15 years), **b** ER-negative tumours and **c** ER-positive tumours. The *y-*axis shows the −log_10_ *P* values of each variant analysed, and the *x*-axis shows their chromosome position. The red horizontal line represents *P* = 5 × 10^−8^

**Fig. 2:**
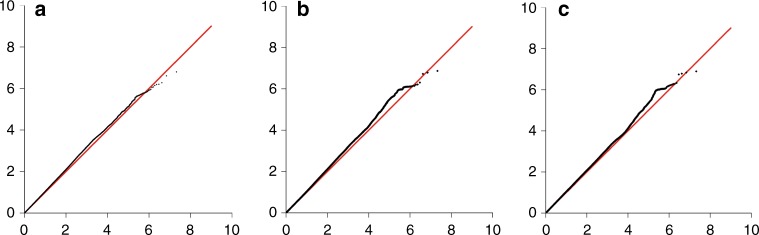
Q–Q plots for the meta-analysis of the twelve datasets for breast cancer-specific mortality analyses (censored at 15 years) for **a** all breast cancer tumours (censored at 15 years), **b** ER-negative tumours and **c** ER-positive tumours. The *y*-axis represents the observed −log_10_ *P* value, and the *x*-axis represents the expected −log_10_ *P* value. The red line represents the expected distribution under the null hypothesis of no association. Analyses were not corrected for LD-structure

Genotype data from 96,661 breast cancer cases (64,171 ER-positive and 16,172 ER-negative) with 7697 breast cancer deaths within 15 years were included in the primary analyses. For 16,318 cases we did not have ER-status information. The average follow-up time was 6.38 years. Details of the numbers of samples and events in each dataset are given in Supplementary Table 3. Manhattan and quantile-quantile (Q–Q) plots for the associations between variants and breast cancer-specific mortality of all invasive, ER-negative and ER-positive breast cancers are shown in Fig. 1 and Fig. 2, respectively. There was some evidence of inflation of the test statistic with an inflation factor of 1.06 for all invasive and ER-positive, and 1.05 for ER-negative including all variants. These Q–Q plots showed no evidence of an association at *P* < 5 × 10^−8^; at less stringent thresholds for significance, there were an increasing number of observed associations for all three analyses (Fig. 2).

We identified three variants at BFDP < 15% associated with breast cancer-specific mortality of patients with ER-negative disease (Table 1). These variants are part of an independent set of 32 highly correlated variants^61^ on chromosome 7q21.1 that were associated at *P* < 5 × 10^−6^ (Supplementary Table 4). The LD matrix between these variants computed based on the 1000 European genomes,^62,63^ and their chromosomal positions, are shown in Supplementary Figure 1. The strongest association was for rs67918676: HR = 1.27; 95% CI = 1.16–1.39; *P* = 1.38 × 10^−7^; risk allele A frequency = 0.12 and BFDP = 11%. The imputation efficiency for this variant was high, with *r*^2^ = 0.99 for all datasets.

**Table 1:** Results of the variants with BFDP < 15% in the meta-analysis of the 12 studies of breast cancer-specific mortality

Subgroup	Variant	Chr	Position	Alt	Ref	Eaf_Ref	HR	LCL	UCL	*P* value	BFDP
ER-negative	rs67918676:27445956:A:AT	7	27445956	AT	A	0.12	1.27	1.16	1.39	1.38 × 10^−7^	0.11
ER-negative	rs192185001:27448012:A:AT	7	27448012	AT	A	0.12	1.27	1.16	1.39	1.66 × 10^−7^	0.13
ER-negative	rs145963877:27473909:CAG:C	7	27473909	C	CAG	0.11	1.28	1.17	1.41	1.91 × 10^−7^	0.15
ER-positive	rs4717568:70400700:T:C	7	70400700	C	T	0.62	0.88	0.8	0.92	1.28 × 10^−7^	0.07
ER-positive	rs1917618:70396442:T:A	7	70396442	A	T	0.62	0.88	0.84	0.93	1.46 × 10^−7^	0.08
ER-positive	rs1546774:70398441:T:G	7	70398441	G	T	0.62	0.88	0.84	0.93	1.66 × 10^−7^	0.09
ER-positive	rs1546773:70398437:T:C	7	70398437	C	T	0.62	0.88	0.84	0.93	1.81 × 10^−7^	0.10
All	rs370332736:50395136:AACTT:A	6	50395136	A	AACTT	0.09	1.16	1.10	1.24	2.48 × 10^−7^	0.13

The lead variant rs67918676 is located in an intron of a long intergenic non-coding RNA gene, *LOC105375207* (AC004009.3), in close proximity to the *HOXA* gene cluster and the lncRNA *HOTTIP*. We tested the genes within a 500 MBp window around the 32 highly correlated variants for the association of their mRNA expression in breast tumours with recurrence-free survival using KMplotter (kmplot.com/analysis). Four of the ten closest genes with probes available showed moderate association with breast cancer survival at *P* < 0.005 (*HOXA9*, *HOTTIP*, *EVX1* and *TAX1BP1*), with these associations mainly observed for ER-negative breast cancer (Supplementary Table 5A). Yet, intersecting the germline variants with several sources of genomic annotation information (e.g., chromosome conformation, enhancer–promoter correlations or gene expression) we could not find strong in silico evidence of gene regulation by the region containing the associated variants.

We also identified four variants at a BFDP < 15% associated with breast cancer-specific mortality of patients with ER-positive disease (Table 1). These variants were part of an independent set of 45 highly correlated variants on chromosome 7q11.22 that were associated at *P* < 5 × 10^−6^ (Supplementary Table 6). The LD matrix between these variants computed based on the 1000 European genomes,^62,63^ and their chromosomal positions, are shown in Supplementary Figure 3. The strongest association was for rs4717568: HR = 0.88; 95% CI:0.84–0.92; *P* = 1.28 × 10^−7^; risk allele A frequency = 0.62 and BFDP = 7%. The imputation efficiency for this variant was high, with an average *r*^2^ = 0.96 for all datasets. Two coding genes, *AUTS2* and *GALNT17*, were located within a 500 MBp window around the 45 highly correlated variants, but the expression of neither of the two was associated with breast cancer survival in KMplotter analyses of TCGA data (Supplementary Table 5B).

**Fig. 3:**
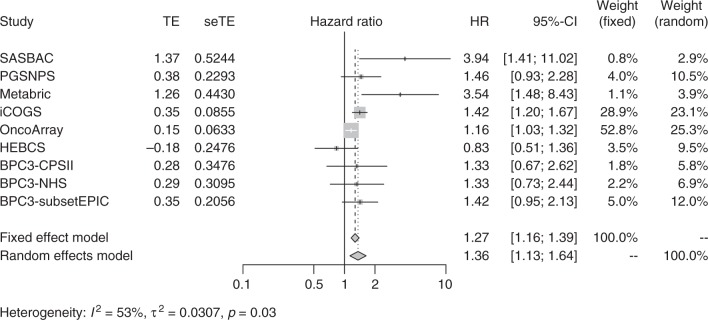
Forest plot showing the association between the ER-negative variant rs67918676 and breast cancer-specific mortality in ER-negative tumours for the datasets used in the meta-analysis. The size of the square reflects the size of the study (see also Supplementary Table 3)

**Fig. 4:**
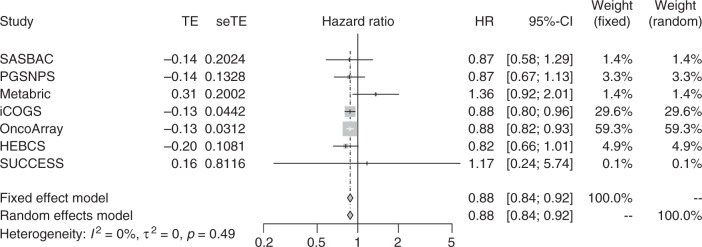
Forest plot showing the association between the ER-positive variant rs4717568 and breast cancer-specific mortality in ER-positive tumours for the datasets used in the meta-analysis. The size of the square reflects the size of the study (see also Supplementary Table 3)

The association of rs67918676 with ER-negative breast cancer was observed in eight of nine studies with no significant heterogeneity present at *P* < 0.01 (Fig. 3 and Supplementary Figure 4a). For ER-positive disease, the association of rs4717568 was detected in all seven studies with no heterogeneity present at *P* < 0.01 (Fig. 4 and Supplementary Figure 4b).

Apart from the 7q variants, only one isolated rare variant reached BFDP values below 15% for all tumours (Table 1). The variant, rs370332736: HR = 1.17; 95% CI: 1.10–1.24; *P* = 2.48 × 10^−7^; risk allele A frequency = 0.09 and BFDP = 13%, is located on chromosome 6 and has an average imputation efficiency of *r*^2^ = 0.96 for all datasets. In addition, there were several variants found at *P* < 10^−6^ for all three analyses (Supplementary Table 4, Supplementary Table 6 and Supplementary Table 7).

## Discussion

In this large survival analysis, we report a genome-wide study for identifying genetic markers associated with breast cancer-specific mortality, involving 96,661 patients from a combined meta-analysis. We found one noteworthy region with 32 highly correlated variants on chromosome 7q21.1 for ER-negative. The lead variant rs67918676 (*P* = 1.38 × 10^−7^ and BFDP of 11% under reasonable assumptions for the prior probability of association) is located in a long intergenic non-coding RNA gene (AC004009.3). While this represents an uncharacterised transcript mainly expressed in testis and prostate, it is located about 200 kb away from a cluster of *HOXA* homeobox genes that has been implicated in breast cancer aetiology and prognosis.^64,65^ This region also contains *HOTTIP*, a lncRNA with prognostic value on clinical outcome in breast cancer.^66^ The flanking region on the opposite side contains *TAX1BP1*, a gene that may be involved in chemosensitivity.^67^ Interestingly, database mining using KMplotter revealed evidence for an association of the expression of these nearby genes with survival from ER-negative breast cancer. On the other hand, the enhancer activity at this noteworthy locus was predicted to be low based on the intersection with biofeatures characteristic of regulatory activity as no known eQTLs appear to exist in this region, suggesting that gene regulatory effects of the identified variants are limited in breast tissue or may be activated under certain untested conditions. For ER-positive tumours, we found another noteworthy region with 45 highly correlated variants at *P* < 5 × 10E^−6^ on chromosome 7q11.22. The lead variant rs4717568 (*P* = 1.28 × 10^−7^ and BFDP of 7%) is located between the *AUTS2* and the *GALNT17* genes. *GALNT17* encodes an N-acetylgalactosaminyltransferase that may play a role in membrane trafficking.^68^ *AUTS2* has been implicated in neurodevelopment,^69^ but *AUTS2* overexpression in cancer has also been linked with resistance to chemotherapy and epithelial-to-mesenchymal transition.^70^ It has been postulated that overexpression of *AUTS2* is specific for metastases,^70^ which may be consistent with the inconspicuous gene expression results in the TCGA database.

It is important to note the differences between the present and the previous GWAS study we had undertaken,^44^ the latter done in a much smaller dataset (3632 events versus 7697 events in the current study) that did not include the OncoArray study. The OncoArray study is the largest dataset used in the present meta-analysis and also the study with the highest imputation quality. The two previously reported variants (rs148760487 for all breast cancer tumours and rs2059614 for ER-negative tumours) were not associated with breast cancer-specific mortality in the current analyses (*P* = 1.59 × 10^−3^ and *P* = 5.41 × 10^−4^, respectively). The most likely explanation for this is that the original results were false-positive findings, despite the original association being nominally “genome-wide significant”. The BDFPs for the original reported associations were 54% and 16%, respectively. For the lead variants identified in the present analysis, we tested for differences in the imputation quality between the current and previous analysis. All variants had high imputation quality (~0.99) in the previous study, suggesting that the longer and more complete follow-up together with a higher number of events allowed more robust identification of breast cancer mortality associations. However, there are some weaknesses of the current meta-analysis such as heterogeneity between patient treatment over time and between countries and between datasets with different study designs that should be considered. These limitations, intrinsic to large survival meta-analyses, increase the noise and reduce the power to detect true associations.

In conclusion, we found two novel candidate regions at chromosome 7 for breast cancer survival, credible at a BFDP < 15% and associated with either ER-negative or ER-positive breast cancer-specific mortality. Concerning additional variants, we might still be underpowered to obtain a more comprehensive picture of genomic markers for breast cancer outcome. Overall, the role of germline variants in breast cancer mortality is still unclear^36,37,71^ and additional analyses with larger sample sizes and more complete follow-up including treatments are needed. In addition, alternative methods that integrate multiple data sources such as gene expression, protein–protein interactions or pathway analyses may be used to aggregate the effect of multiple variants with small effects.^72^ Such approaches could increase the power of the analyses while better explaining the underlying biological mechanisms associated with breast cancer mortality.

## Supplementary information

Supplementary Figures and Tables

Supplementary Table 2

Supplementary Table 4

Supplementary Table 6

Supplementary Table 7

Supplementary Methods

Supplementary Script BFDP

## Data Availability

All estimates reported in the paper are available through the BCAC website: http://bcac.ccge.medschl.cam.ac.uk.
