# Supplementary material for: Genome-wide association study of germline variants and breast cancer-specific mortality
Source: Br J Cancer. 2019 Feb 21;120(6):647–57. doi: 10.1038/s41416-019-0393-x (PMC6461853; doi:10.1038/s41416-019-0393-x)

**Supplementary Data**

**List of Tables**

**Supplementary Table 1.** Description of the twelve datasets by genotyping platform

**Supplementary Table 2.** Data design of the studies included in the analysis

**Supplementary Table 3.** Follow-up and ER-status description for the twelve datasets

**Supplementary Table 4.** Results of top 100 variants in the combined analysis of ER-negative breast cancer-specific mortality

**Supplementary Table 5.** Association of survival with gene expression of nearby genes at the chr7q21.1 locus (A) and chr7q11.22 locus (B)

**Supplementary Table 6.** Results of top 100 variants in the combined analysis of ER-positive breast cancer-specific mortality

**Supplementary Table 7.** Results of top 100 variants in the combined analysis of all breast cancer-specific mortality

**List of Figures**

**Supplementary Figure 1.** Kaplan-Meier Curves for the cohorts used in the meta-analysis

**Supplementary Figure 2.** Linkage disequilibrium matrix for the ER-negative variants associated with breast cancer-specific mortality in ER-negative tumours

**Supplementary Figure 3.** Linkage disequilibrium matrix for the ER-positive variants associated with breast cancer-specific mortality in ER-positive tumours

**Supplementary Figure 4.** Forest plots showing the association between breast cancer specific-mortality and the most significant variant in ER-negative and ER-positive disease for iCOGS and OncoArray separately: by study in iCOGS and by country in OncoArray. (a) ER-negative most significant variant: rs67918676. (b) ER-positive most significant variant: rs4717568. The size of the square reflects the size of the study

**Supplementary Table 1.** Description of the twelve datasets by genotype platform

| **Study** | **Genotyping platform** | **Number of principal components*** | **Age at diagnosis in years** | **Sample size** | **Country** | **Description of the study** |
| --- | --- | --- | --- | --- | --- | --- |
| OncoArray | Illumina OncoArray-500K BeadChip | 10 | 18-98 | 54,798 | International | Breast cancer patients from 62 studies of the Breast Cancer Association Consortium (BCAC), genotyped as part of the OncoArray Consortium^1^ |
| COGS | Illumina iSelect | 9 | 16-96 | 29,959 | International | Breast cancer patients from 34 studies of the BCAC, genotyped as part of the Collaborative Oncological Gene-Environment Study (COGS)^2^ |
| CGEMS | Illumina Hap550K | 0 | 44-83 | 1,145 | USA | Breast cancer patients from Nurses Health Study genotyped as part of CGEMS project^3^ |
| SASBAC | Illumina HumanHap300 and HumanHap240S | 0 | 50-75 | 787 | Sweden | Breast cancer patients from Swedish Case-control study, part of BCAC^4^ |
| UK2 | Illumina 670k | 3 | 17-88 | 2,763 | UK | Consist of National study of breast cancer of age < 41 years, and Subset of samples from national familial breast cancer study^5^ |
| Metabric | Affymetrix SNP 6.0 | 1 | 26-96 | 369 | UK | UK samples from international breast cancer genomics project^6^ |
| PG-SNPs | Affymetrix SNP 6.0 | 2 | 22-77 | 1,797 | UK | UK samples from breast cancer chemotherapy treatment response study^7–10^ |
| HEBCS | Illumina 550K | 0 | 26-87 | 742 | Finland | Helsinki Breast Cancer Study^11–14^ |
| SUCCESS-A | Illumina HumanOmniExpress-12v1 FFPE | 0 | 19-85 | 3,229 | Germany | A Genome-Wide Association Study in Breast Cancer Patients From the Prospectively Randomized SUCCESS Trial^15^ |
| BPC3-CPSII | Illumina 660W | 0 | 51-89 | 293 | USA | The National Cancer Institute Breast and Prostate Cancer Cohort Consortium: American Cancer Society Cancer Prevention Study-II^16^ |
| BPC3-EPIC | Illumina 660W | 0 | 27-75 | 476 | Europe | The National Cancer Institute Breast and Prostate Cancer Cohort Consortium: European Prospective Investigation of Cancer^16^ |
| BPC3-NHS2 | Illumina 550K | 0 | 44-83 | 233 | USA | The National Cancer Institute Breast and Prostate Cancer Cohort Consortium: Nurses' Health Studies II^16^ |

*****Number of principal components included in the survival analyses to correct for population structure^17^

**Supplementary Table 3.** Follow-up and ER-status description for the twelve datasets

| **Dataset** | **All tumours** | | **ER-positive** | | **ER-negative** | |
| --- | --- | --- | --- | --- | --- | --- |
|  | **N (breast cancer deaths)** | **Person-years** | **N (breast cancer deaths)** | **Person-years** | **N (breast cancer deaths)** | **Person-years** |
| OncoArray | 54,798 (3,632) | 346,059 | 38,685 (2,159) | 233,298 | 8,424 (1,048) | 52,507 |
| COGS | 29,959 (2,643) | 196,439 | 20,249 (1,441) | 132,676 | 4,775 (614) | 29,790 |
| CGEMS | 1,145 (93) | 7,711 | -- | -- | -- | -- |
| SASBAC | 787 (89) | 4,133 | 483 (53) | 2,539 | 108 (15) | 551 |
| UK2 | 2,763 (305) | 29,664 | -- | -- | -- | -- |
| Metabric | 369 (88) | 1,582 | 291 (61) | 1,280 | 63 (25) | 225 |
| PG-SNPs | 1,797 (211) | 5,884 | 1,192 (122) | 3,957 | 591 (87) | 1,906 |
| HEBCS | 742 (321) | 5,652 | 492 (197) | 4,202 | 196 (106) | 1,214 |
| SUCCESS-A | 3,299 (175) | 13,145 | 2264 (83) | 9,289 | 1,013 (90) | 3,806 |
| BPC3-CPSII | 293 (30) | 2,544 | -- | -- | 293 (30) | 2,544 |
| BPC3-EPIC | 476 (74) | 2,226 | -- | -- | 476 (74) | 2,226 |
| BPC3-NHS2 | 233 (36) | 2,732 | -- | -- | 233 (36) | 2,732 |
| **Total** | **96,661 (7,697)** | **622,404** | **64,171 (4,116)** | **424,377** | **16,172 (2,125)** | **133,365** |

ER=estrogen receptor

**Supplementary Table 5A:** Association of survival with gene expression of nearby genes at the chr7q21.1 locus

| **Study Group** | ***HOXA9*** (209905_at) | ***HOXA10-AS*** (231365_at) | ***HOXA10*** (213150_at) | ***HOXA11*** (213823_at) | ***HOXA11-AS*** (230666_at) | ***HOXA13*** (231786_at) | ***HOTTIP*** (244553_at) | ***EVX1*** (207914_x_at) | ***HIBADH*** (234452_at) | ***TAX1BP1*** (213786_at) |
| --- | --- | --- | --- | --- | --- | --- | --- | --- | --- | --- |
| All | 1.12 (1.00-1.25), p=0.059 | 0.90 (0.77-1.06), p=0.2 | 0.88 (0.79-0.99), p=0.032 | 0.88 (0.79-0.99), p=0.032 | 0.81 (0.69-0.95), p=0.0097 | 1.18 (1.01-1.39), p=0.042 | 0.79 (0.67-0.92), p=0.0036 | 0.76 (0.68-0.85), p=2.4x10E-6 | 0.93 (0.79-1.09), p=0.36 | 1.21 (1.08-1.36), p=0.00098 |
| ER-negative  (GE-based) | 1.38 (1.11-1.72), **p=0.0042** | 1.16 (0.88-1.53), p=0.29 | 1.04 (0.83-1.29), p=0.74 | 0.94 (0.75-1.17), p=0.58 | 0.93 (0.70-1.22), p=0.58 | 1.10 (0.84-1.45), p=0.5 | 0.67 (0.51-0.89), **p=0.0047** | 0.68 (0.54-0.85), **p=0.00065** | 1.00 (0.76-1.32), p=0.99 | 1.41 (1.13-1.76), **p=0.0023** |
| ER-positive  (GE-based) | 1.04 (0.91-1.18), p=0.6 | 0.77 (0.63-0.94), p=0.0091 | 0.84 (0.74-0.96), p=0.012 | 0.87 (0.76-1.00), p=0.042 | 0.73 (0.60-0.89), p=0.0018 | 1.17 (0.96-1.43), p=0.12 | 0.82 (0.67-1.00), p=0.046 | 0.80 (0.70-0.92), p=0.0012 | 0.89 (0.73-1.09), p=0.25 | 1.15 (1.00-1.31), p=0.045 |

**Supplementary Table 5B:** Association of survival with gene expression of nearby genes at the chr7q11.22 locus

| **Study Group** | ***AUTS2*** (243364_at) | ***GALNT17*** (227434_at) |
| --- | --- | --- |
| All | 0.89 (0.76-1.04), p=0.13 | 0.94 (0.81-1.10), p=0.45 |
| ER-negative  (GE-based) | 0.81 (0.62-1.06), p=0.12 | 1.17 (0.94-1.52), p=0.24 |
| ER-positive  (GE-based) | 0.87 (0.65-1.16), p=0.35 | 0.84 (0.70-1.02), p=0.08 |

Legend: Genes with available probes within a 500 MBp window centred at the identified set of highly correlated variants and were tested for the association of their mRNA/ncRNA expression in breast tumours with recurrence-free survival using KMplotter (kmplot.com/analysis). The headers of the columns indicate the genes tested and the gene expression probes used. Association analyses were performed for all breast cancer, ER-negative breast cancer and ER-positive breast cancer, with ER status based on gene expression. Hazard ratios are shown with 95% confidence intervals. A. Results for chr7q21.1 locus, p-values<0.005 for ER-negative breast cancer are indicated in bold. B. Results for chr7q11.22 locus in ER-positive breast cancer. ER=estrogen receptor; GE=gene expression.

**Supplementary Figure 1.** Kaplan-Meier Curves for the cohorts used in the meta-analysis*

**
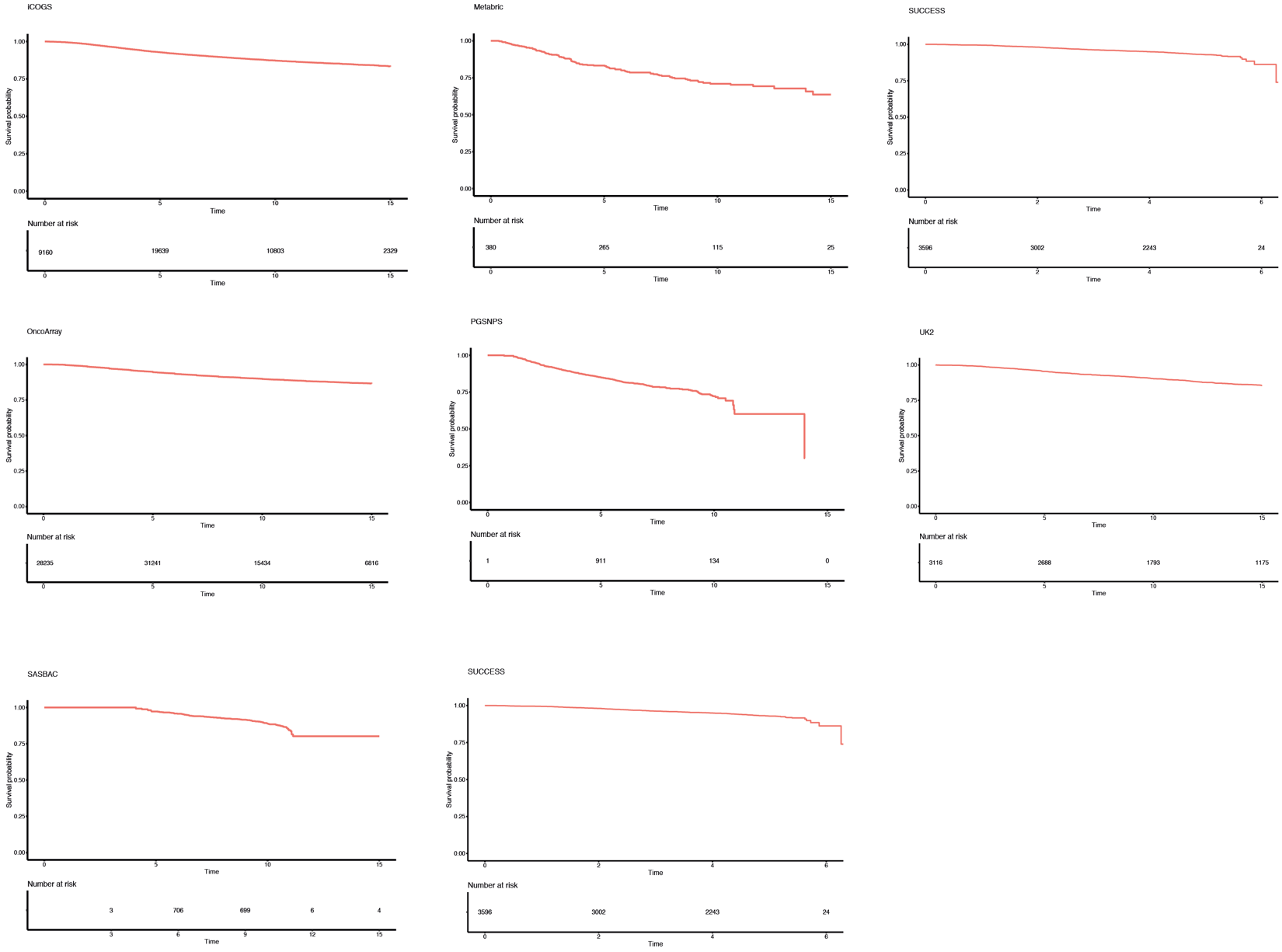
**

* We did not have individual patient survival data for the CGEMS, HEBCS and BPC3 studies (only summary estimates were available to BCAC)

**Supplementary Figure 2.** Linkage disequilibrium matrix for the ER-negative variants associated with breast cancer-specific mortality in ER-negative tumours

**Supplementary Figure 3.** Linkage disequilibrium matrix for the ER-positive variants associated with breast cancer-specific mortality in ER-positive tumours

**Supplementary Figure 4.** Forest plots showing the association between breast cancer specific-mortality and the most significant variant in ER-negative and ER-positive disease for iCOGS and OncoArray separately: by study in iCOGS and by country in OncoArray. (a) ER-negative most significant variant: rs67918676. (b) ER-positive most significant variant: rs4717568. The size of the square reflects the size of the study


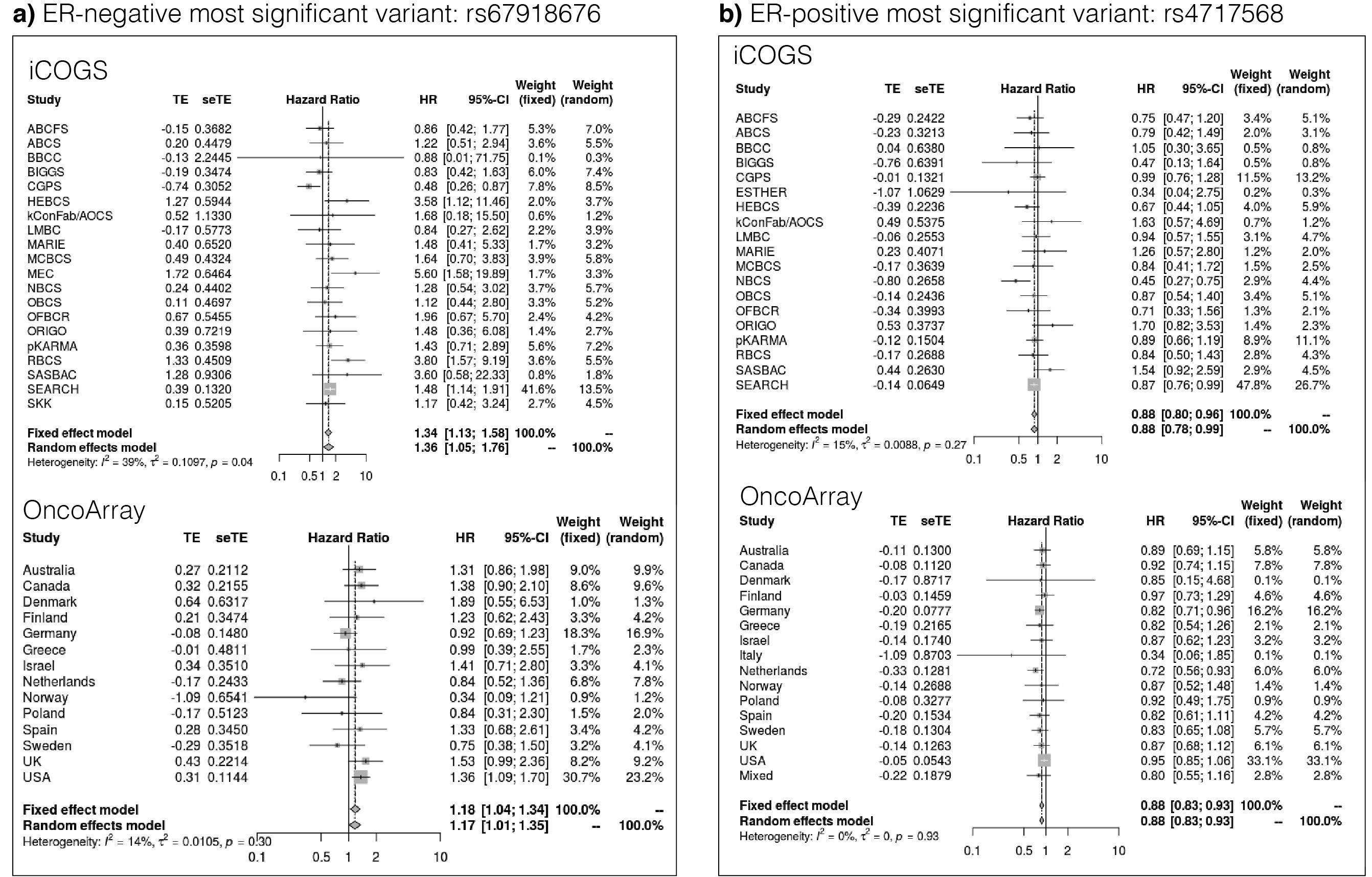

Supplement: Supplementary file 1 — Supplementary Figures and Tables [file 41416_2019_393_MOESM1_ESM.docx]
