# Supplementary material for: Genome-wide association study of germline variants and breast cancer-specific mortality
Source: Br J Cancer. 2019 Feb 21;120(6):647–57. doi: 10.1038/s41416-019-0393-x (PMC6461853; doi:10.1038/s41416-019-0393-x)
Supplement: Supplementary file 6 — Supplementary Methods [file 41416_2019_393_MOESM6_ESM.docx]

**Supplementary methods**

Derivation of the Standard Errors (SE) for maximum-likelihood estimates using Likelihood Ratio Test Statistics.

The likelihood ratio test (LRT) and the Wald test are identical for normally distributed data (where the log-likelihood is quadratic) and also asymptotically equivalent (i.e. approximately equivalent in large samples; see for example Ch.9 of Cox and Hinkley’s *Theoretical Statistics* (1974)^67^). In finite samples the LRT is generally preferable – in particular it has better properties for association tests based on rare counts, and we have therefore used LRT and maximum-likelihood estimates of all datasets.

In the meta-analysis the test statistic is of the form:

$$\frac{\sum Z_{j}\sqrt{w_{j}}}{\sqrt{\sum w_{j}}}$$

and the summary estimate is of the form:

$$\frac{\sum w_{j}\theta_{j}}{\sum w_{j}}$$

where $\theta_{j}$are the study-specific estimates, $Z_{j}$are the study-specific signed test statistics, and $w_{j}$are study-specific weights. The meta-analysis is valid whatever weights are used but the most efficient test uses inverse variance weights, and hence requires approximate standard errors for the parameter estimates. To derive approximate standard-errors for the maximum likelihood estimates, we can use the asymptotic equivalence of the LRT and the Wald test.

The likelihood ratio statistic (LRT) is of the form:

$$W=2\{l\left( \hat{\theta};Y \right)-l\left( \theta_{0};Y \right)\}$$

where $\theta_{0}$ is the null value of the parameter (0 in this case), $\hat{\theta}$ is the maximum likelihood estimate (MLE) and $l\left( \theta_{0};Y \right)$ is the log-likelihood given the data *Y*.

Expanding in a Taylor’s series (Cox and Hinkley’s pp. 313):

$$W=2\left( \hat{\theta}-\theta_{0} \right)U\left( \hat{\theta} \right)-\left( \hat{\theta}-\theta_{0} \right)^{2}U'\left( \theta^{+} \right)$$

$=-\left( \hat{\theta}-\theta_{0} \right)^{2}U'\left( \theta^{+} \right)$ (1)

where $U$ refers to the first derivative of the log-likelihood and $U$’ the second derivative, since by definition $U\left( \hat{\theta} \right)=0$ at the maximum-likelihood, where $\left| \theta^{+}-\theta_{0} \right|<\left| \theta-\theta_{0} \right|$.

Since, asymptotically, $\hat{\theta}$ is consistent, $U'\left( \theta^{+} \right)\approx U'\left( \hat{\theta} \right)$

The variance of $\hat{\theta}$ is, asymptotically, $1/i\left( \theta\right)=-1/E(U^{'}\left( \theta\right);\theta)$, that is the inverse of the information matrix (Cox and Hinkley pp. 294). Hence this can be estimated by $-1/U^{'}\left( \hat{\theta} \right)$ (minus the second derivative of the log-likelihood at the MLE).

Combining with (1), the variance (and hence standard error) of the maximum likelihood estimate $\hat{\theta}$, can thus be estimated, using the likelihood ratio test statistic W, by:

$$var(\hat{\theta})=\left( \hat{\theta}-\theta_{0} \right)^{2}/W$$
